# Comparative Evaluation of Fracture Resistance and Failure Modes in Endodontically Treated Teeth Restored With Zirconia-Reinforced Lithium Silicate and Cast Metal Alloys: An In Vitro Study

**DOI:** 10.7759/cureus.77727

**Published:** 2025-01-20

**Authors:** Satheesh R, Rekha Gupta, Shubhra Gill, Akshita Mehta

**Affiliations:** 1 Department of Prosthodontics, Maulana Azad Institute of Dental Sciences, New Delhi, IND

**Keywords:** endocrown, one-piece dowel crown, post & core, post endo restoration, richmond crown, zirconia reinforced lithium silicate

## Abstract

Introduction

This study evaluates the mechanical performance of a new all-ceramic material, zirconia-reinforced lithium silicate (ZLS), as a restorative material for the rehabilitation of endodontically treated teeth with severe coronal destruction.

Material and methods

Sixty extracted mandibular first premolars of similar size and shape were collected, and root canal treatment and tooth preparation were performed. They were divided into six groups as follows: Group 1 A - ZLS post and core, Group 1 B - ZLS one-piece dowel crown, Group 1 C - ZLS endocrown, Group 2 A - Cast post & core with metal crown, Group 2 B - Cast one-piece dowel crown, Group 2 C - Metal endocrown. After etching and bonding, all restorations were cemented with dual-cure resin cement. All specimens were loaded until failure in a Universal Testing Machine. Fracture analysis and failure modes analysis were conducted through visual examination. For statistical analysis, a one-way ANOVA test was used to analyze fracture resistance values.

Results

Within Group 1, the mean fracture resistance was highest for the ZLS post and core (610 N), followed by the ZLS endocrown (450 N) and the ZLS one-piece dowel crown (356 N), respectively, and this difference was statistically significant (P < 0.05). An inter-group comparison using an unpaired t-test showed a significant difference between the ZLS post and core (1A) and the Cast post and core (2A) (P < 0.05).

Conclusion

ZLS restorations have great potential to be used as an alternative to cast metal restorations for the restoration of severely damaged endodontically treated teeth.

## Introduction

Endodontically treated teeth are fraught with significant loss of structural integrity, accompanied by diminished moisture and collagen content, rendering them inherently brittle. These vulnerabilities pose a challenge in their effective rehabilitation [1]. Rehabilitation of an endodontically treated tooth includes various dowel and retention systems, from traditional post and core to Richmond crowns and endocrowns, with modern adhesive techniques broadening treatment options. Restoration materials range from custom-cast post and core to computer-aided design and computer-aided manufacturing (CAD-CAM) fabricated zirconia posts [2]. The choice of post material and design is crucial for the durability of both the tooth and the restoration. Custom fabricated cast posts have been used in endodontically treated teeth but are non-aesthetic and carry a risk of root fracture [3]. Ceramic materials like glass ceramic, feldspathic ceramic, aluminum oxide, and zirconia are used for post and core restorations, offering excellent aesthetics but being brittle, costly, and technique-sensitive [4]. The Richmond crown, introduced by Richmond TW [5] in 1878, initially featured a threaded tube in the canal with a screw-retained crown, later modified into a one-piece dowel crown. Even though it is not the first treatment choice, it remains suitable for cases with deep bites, minimal overjet, or limited interocclusal space. Limitations include excessive tooth preparation to align two axes, increasing stress at the post apex, acting as a wedge under occlusal load, and being time-consuming [5].

The endocrown, introduced by Bindl A and Mormann WH [6] in 1999, is a monolithic ceramic adhesive restoration that requires a cervical butt joint and pulp chamber preparation, without involving the root canals. Its benefits include minimal tooth reduction, reduced procedure time, and a simpler technique. However, it is not appropriate for teeth with short or narrow pulp chambers [7, 8]. 

Zirconia-reinforced lithium silicate (ZLS) is a new material combining the optical properties of lithium disilicate with the mechanical strength of zirconia [9].

Selecting the appropriate material and restoration type is often a challenging decision. This study has been formulated to evaluate ZLS-based restorations (Post & Core, One-piece Dowel Crown, and Endocrown) against traditional cast metal alloys for fracture resistance and failure modes. A null hypothesis has been posited that there is no difference between ZLS and cast metal alloys in terms of fracture resistance and failure modes.

## Materials and methods

Based on data from previous studies, statistical analysis was conducted, and the number of specimens required in each test group was determined to be 10. Thus, a total of sixty extracted single-rooted mandibular first premolars without fractures, pre-existing restorations, or developmental anomalies, and with similar size and shape, were collected.

Recently extracted teeth (less than 2 months) were collected and cleaned by scrubbing to remove adherent particulate material, followed by storage in securely sealed plastic containers with a 1:10 sodium hypochlorite solution [10]. A silicone index of the anatomic crown below the cementoenamel junction (CEJ) was made before any preparation, which will help in the fabrication of patterns for the crown. After the removal of unsupported enamel, the teeth's anatomic crowns were sectioned parallel to the CEJ under water with a diamond disc on a straight handpiece, leaving at least 2 mm of sound structure above the CEJ to provide the ferrule [11]. For the remainder of the investigation, the specimens were kept at room temperature in a 0.9 percent saline solution [11]. Standard root canal procedures were performed on all teeth, with a working length of 0.5 mm from the radiographic apex. Using the step-back technique, the root canals were manually instrumented to an apical size of ISO 40. The canals were dried with absorbent paper points and obturated with gutta-percha (M-access, Dentsply, Germany) and resin-based sealer (AH Plus root canal sealer, Dentsply), using the cold lateral condensation technique. Endodontic treatment of all the teeth was done by a single operator for standardization. After endodontic treatment, periodontal ligament (PDL) simulation was done with addition silicone impression material (President, Coltene, Switzerland), followed by embedding in auto-polymerizing acrylic resin (DPI-RR, India) at a level 2 mm below the CEJ with the help of a surveyor for vertical alignment of the tooth in the acrylic resin. Tooth preparation for post and core and one-piece dowel crown are similar.

**Figure 1:**
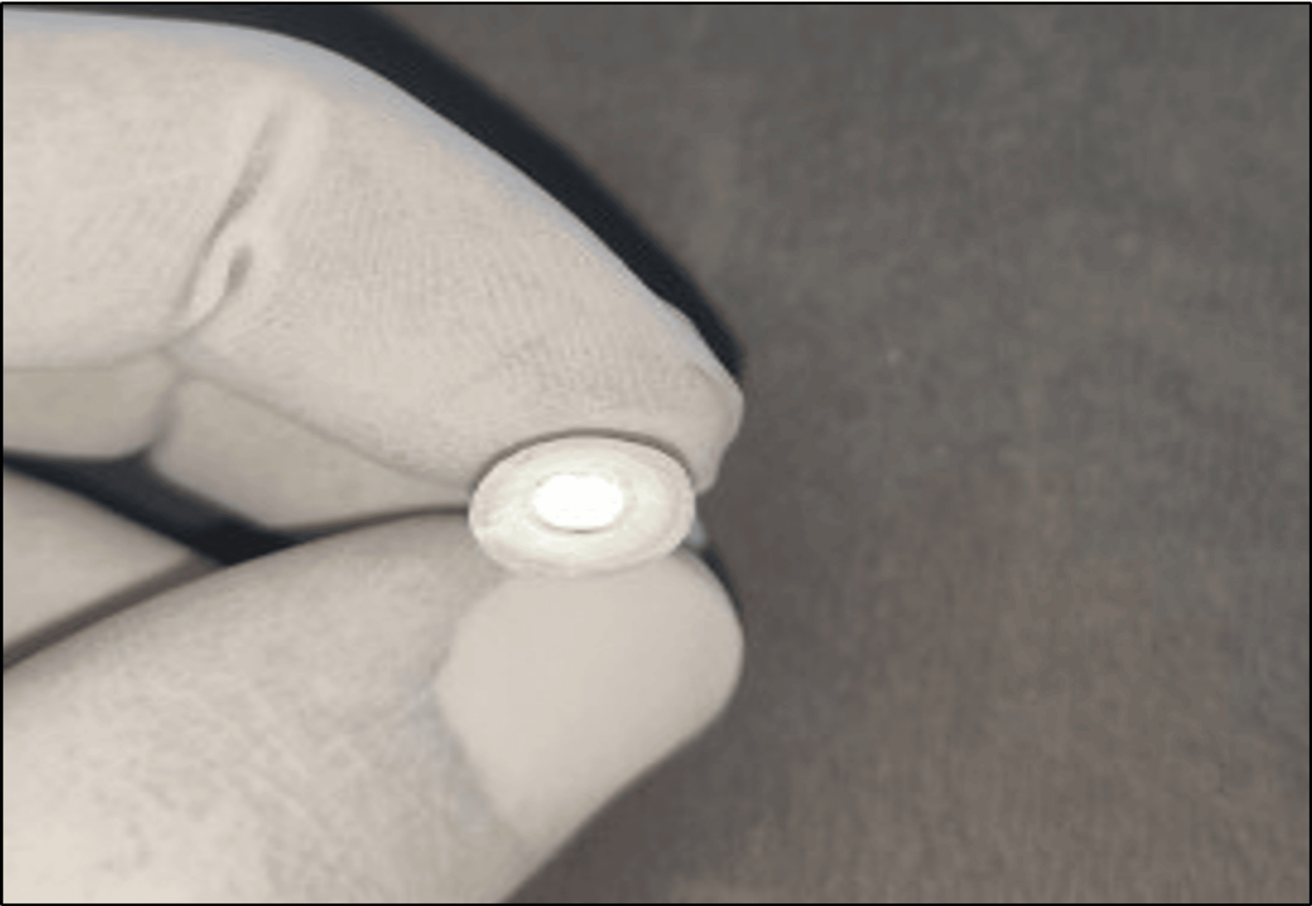
Tooth preparation for endocrown.

After root canal treatment (RCT), gutta-percha was removed using a Peeso reamer (MANI, Japan) without disturbing the apical seal. An apical seal of at least 4 mm was maintained. Post space preparation was done with a Peeso reamer size 4. A radial shoulder finish line of 1 mm width and rounded internal line angles was prepared to provide a ferrule height of 2 mm. For the endocrown tooth preparation, the occlusal surfaces were made flat by a diamond wheel bur oriented parallel to the occlusal plane to ensure a flat surface, which determines the position of the cervical margin or ‘cervical sidewalk’. The cervical margin was placed supragingivally. Enamel walls less than 2 mm thick were eliminated since they are prone to fracture due to reduced structural durability. All teeth in this group underwent a uniform cavity preparation that includes removing undercut portions of the pulp chamber and aligning its axial walls with an internal taper of around 8-10 degrees to provide retention and ease of insertion. A tapered diamond bur with a rounded end held perpendicular to the pulpal floor was used for providing the internal taper. All internal line angles were smoothed down and rounded (Figure 1). To make use of the cavity floor's saddle-like architecture, gutta-percha was removed to a depth of no more than 2 mm.

**Figure 2:**
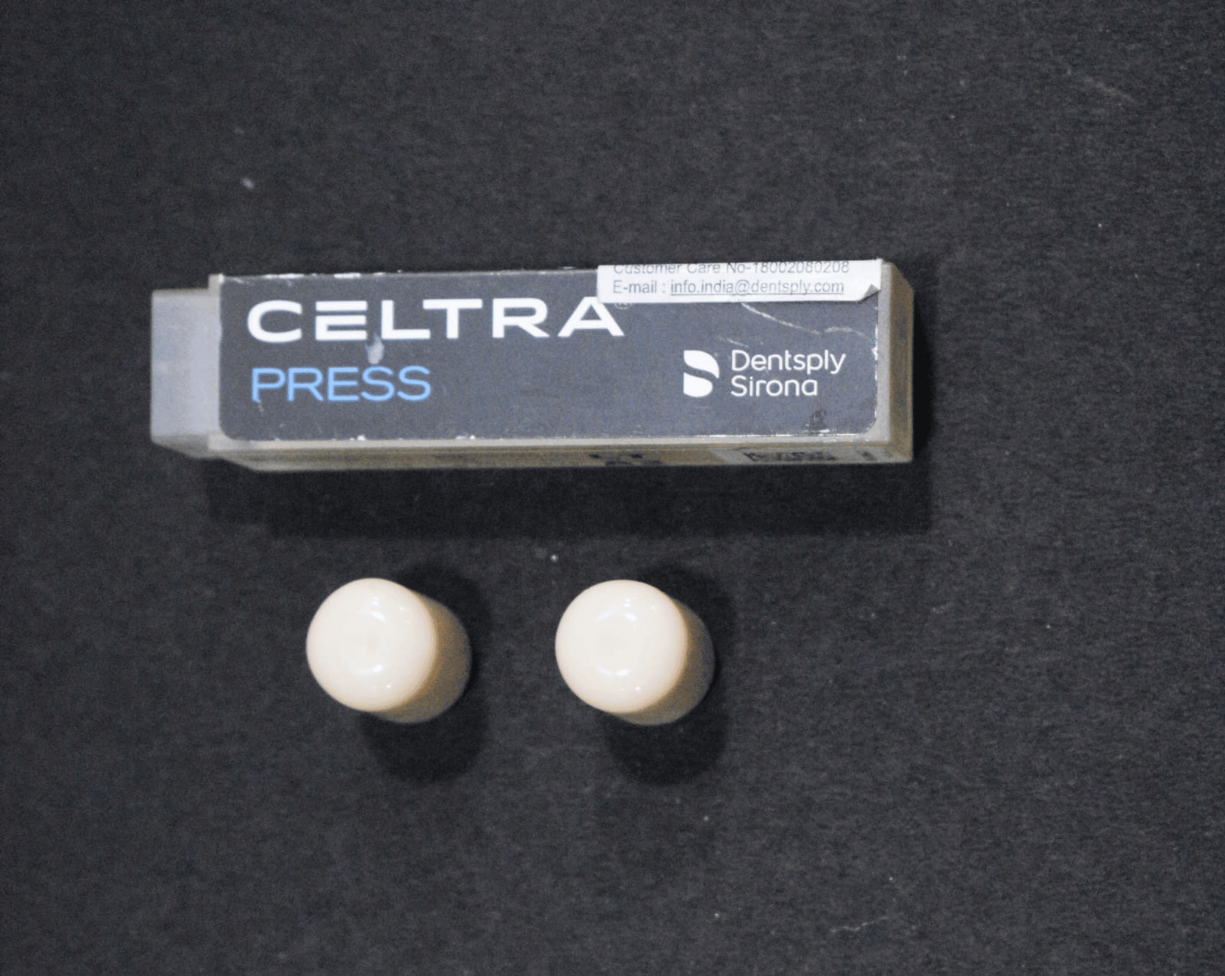
ZLS Celtra press blocks. ZLS: Zirconia-reinforced lithium silicate.

Now, the sixty samples are divided into two main groups of 30 specimens each. Group 1 samples are restored with new all-ceramic material ZLS (Celtra Press blocks, Dentsply Sirona, Germany) and Group 2 samples are restored with conventional cast metal alloys (Figure 2). Group 1 and Group 2 are again subdivided into three subgroups based on the restorative design used. Now there are 6 groups as follows: Group 1A - ZLS post and core with crown, Group 1 B - ZLS one-piece dowel crown, Group 1C - ZLS endocrown, Group 2A - cast post and core with metal crown, Group 2B - cast one-piece dowel crown, Group 2C - Metal endocrown.

**Figure 3:**
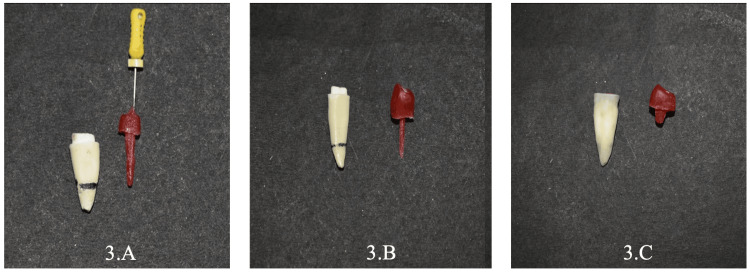
3.A: Patterns of post & core; 3.B: One-piece dowel crown; 3.C: Endocrown preparations recorded with pattern resin.

**Figure 4:**
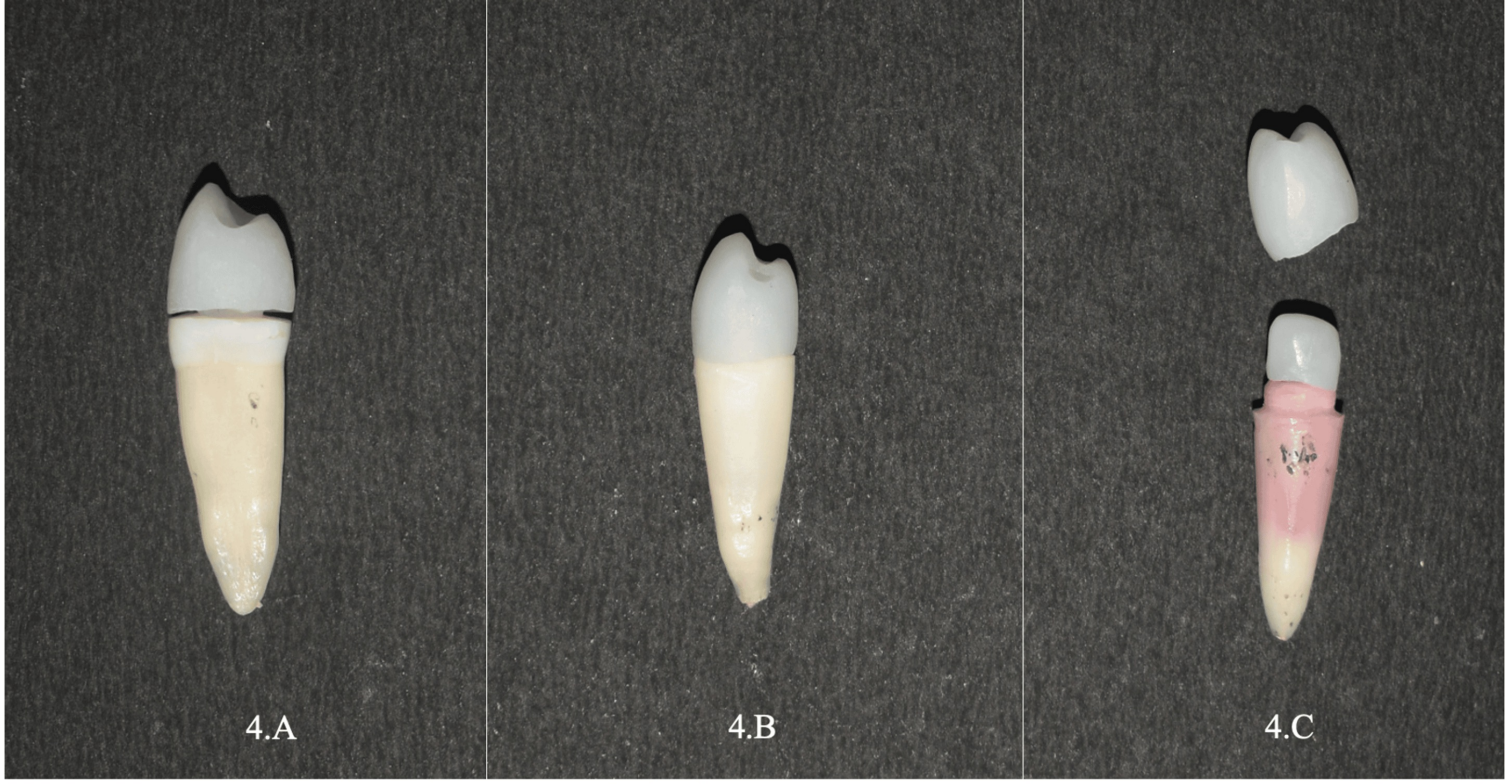
4.A: Fabricated ZLS endocrown; 4.B: One-piece dowel crown; 4.C: Post & core restorations.

For the fabrication of ZLS post and core restorations (Group 1A), the post space was recorded with pattern resin (GC Corporation, Tokyo, Japan) and the pattern was pressed in an IPS Emax pressing unit, and the ZLS post and core was fabricated (Figures 3-4). For the fabrication of ZLS one-piece dowel crowns (Group 1B), the post space was recorded with pattern resin and the pattern was pressed in an IPS Emax pressing unit and the ZLS one-piece dowel crown was fabricated (Figures 3-4). For the fabrication of ZLS endocrowns (Group 1C), the preparation space and the margins were recorded by pattern resin and with the help of this pattern, ZLS endocrown was fabricated using an IPS Emax pressing unit (Figures 3-4).

For the fabrication of cast post and core with metal crown (Group 2A), the post space was recorded with pattern resin and the pattern was invested and cast to form the cast post and core over which a metal crown was fabricated. For the fabrication of cast one-piece dowel crowns (Group 2B), the post space was recorded with pattern resin and the pattern was invested and cast to form the cast one-piece dowel crown. For the fabrication of metal endocrowns (Group 2C), the preparation space and the margins were recorded by pattern resin and with the help of this pattern, the metal endocrown was fabricated. For preparing the patterns for the crowns, the silicone putty index which was made before tooth sectioning was used for standardization. The post and core group were restored with complete coverage crowns to ensure standardization and to replicate the clinical situation. The ZLS post and core group received full coverage ZLS crowns while the cast post and core group received full coverage cast metal crowns.

**Figure 5:**
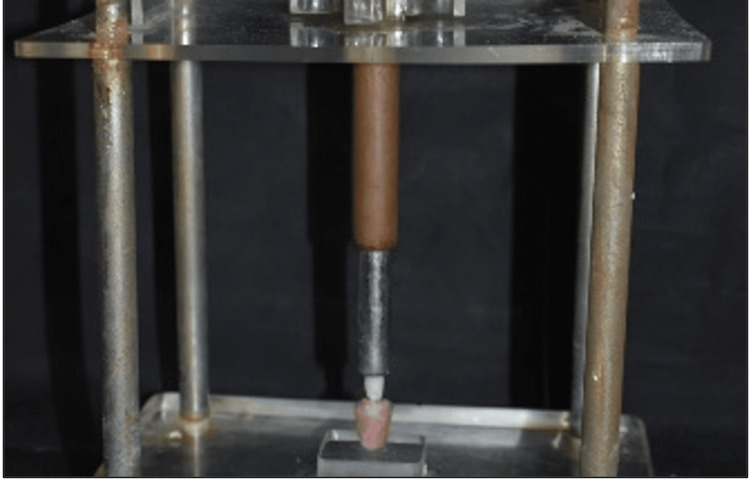
Restoration cemented using a universal alignment apparatus.

After the fabrication of all restorations, the post space and the prepared spaces of all teeth were etched with 37% orthophosphoric acid (Actino gel, Prevest Denpro, USA) for 15 seconds, and the bonding agent (Calibra Esthetic Resin Cement Kit; Dentsply Sirona) was applied and cured for 10 seconds. Now the ZLS restorations were also etched with 9.6% hydrofluoric acid (Angelus, Brazil) for 60 seconds and a silane coupling agent (Dentsply Sirona) was applied and left to dry out. Then dual cure resin cement (Calibra Esthetic Resin Cement Kit; Dentsply Sirona) is manipulated according to manufacturer instructions and placed inside the preparation and ZLS restorations were luted with the help of a universal alignment apparatus with a load of 1 kg and kept for about 10 minutes for standardization (Figure 5). Cast metal restorations were sandblasted (Macro Dental, India) and a metal primer (Alloy Primer, Kuraray Co Ltd, Japan) applied and allowed to dry out. Then dual cure resin cement (Calibra Esthetic Resin Cement) was manipulated according to manufacturer’s instructions and placed inside the preparation and cast metal restorations were luted with the help of a universal alignment apparatus with a load of 1 kg and kept for about 10 minutes for standardization.

**Figure 6:**
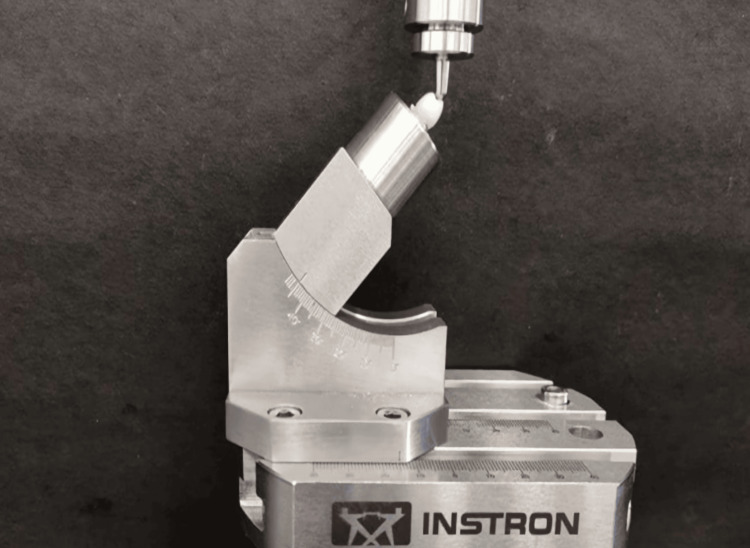
Fracture loading of ZLS post & core in a Universal Testing Machine.

All specimens were loaded until failure in a Universal Testing Machine (Instron, USA) after cementation (Figure 6). The specimens were positioned at a 45-degree angle to the tooth's long axis. The crosshead moved at a rate of 0.5 millimeters per minute. The force was applied with the help of a metal plunger with a diameter of 4 mm, midway between the lingual slope of the buccal cusp and the central fissure [2].

**Figure 7:**
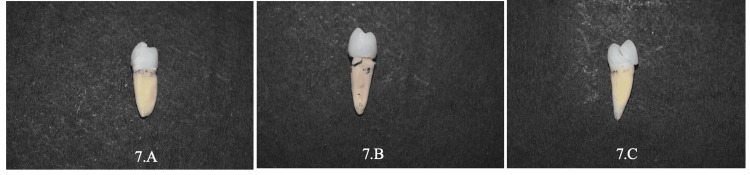
Failure of ZLS crown. 7.A: Non-catastrophic failure of ZLS post & core; 7.B: Catastrophic failure of ZLS one-piece dowel crown; 7.C: Non-catastrophic failure of ZLS endocrown. ZLS: Zirconia-reinforced lithium silicate.

**Figure 8:**
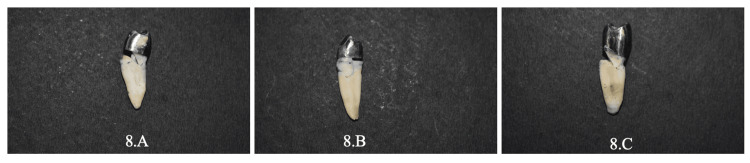
Failure of castable crown. 8.A: Catastrophic failure of cast post & core; 8.B: Catastrophic failure of cast one-piece dowel crown; 8.C: Catastrophic failure of cast endocrown.

The load was applied until fractures occurred. The fracture load was recorded in Newtons (N) [12]. This abrupt drop was interpreted as a crack or fracture in the sample, and the maximal force up to this point was recorded as the force of fracture. The positions of cracks, fragmentation of core components or teeth, and debonding were all analyzed visually (Figures 7-8).

The following patterns were used to categorize the failure modes: Non-catastrophic failures are repairable fractures of teeth above the CEJ level, while catastrophic failures are non-repairable fractures of teeth below the CEJ level. The data for fracture resistance was collected after fracture loading in the Universal Testing Machine. The information was entered into an Excel spreadsheet and analyzed with the SPSS Inc., Chicago, IL, USA, version 26. Results were considered significant when a p-value of less than 0.05 was derived (confidence interval of 95%). In this study, descriptive and inferential statistics were calculated. 

## Results

The null hypothesis, which states that there is no difference in the fracture resistance and failure modes of severely damaged endodontically treated teeth restored with ZLS and cast metal alloys, is rejected due to a significant difference in fracture resistance between ZLS restorations and cast metal alloy restorations. Table 1 shows descriptive statistics of mean fracture resistance for each group. Overall, the mean fracture resistance is highest for ZLS post & core (610 N), followed by Cast post & core (488 N), and least for ZLS one-piece dowel crown (356 N). In the intra-group comparison of fracture resistance among ZLS restorations, a one-way ANOVA showed a p-value of 0.003 (p < 0.05), indicating a statistically significant difference between the three different ZLS groups (Table 2). Further, Tukey’s post hoc for multiple comparisons showed that the mean fracture resistance for ZLS post & core (group 1.A) was statistically significantly different from ZLS one-piece dowel crown (group 1.B) with a p-value of 0.002 (p < 0.05) and ZLS endocrown (group 1.C) with a p-value of 0.0054 (p < 0.05). However, there was no significant difference between ZLS one-piece dowel crown (group 1.B) and ZLS endocrown (group 1.C).

**Table 1:** Descriptive statistics of mean fracture resistance for each group.

Group	Sample Size (n)	Mean Fracture Resistance (N)	SD	Minimum	Maximum
1.A-Zirconia-Reinforced Lithium Silicate Post & Core	10	610.2110	191.96628	347.55	1048.43
1.B-Zirconia-Reinforced Lithium Silicate One Piece Dowel Crown	10	356.0430	84.00511	238.56	531.89
1.C-Zirconia-Reinforced Lithium Silicate Endocrown	10	450.1780	148.67770	225.90	784.74
2.A-Cast Post & Core	10	482.3680	61.40558	356.17	562.39
2.B-Cast One Piece Dowel Crown	10	488.5540	160.99059	224.44	645.14
2.C-Metal Endocrown	10	378.2370	187.83843	80.80	752.72

**Table 2:** Intra-group comparison of fracture resistance among Group 1.A, 1.B, and 1.C (ZLS) by one-way ANOVA. ZLS: Zirconia-reinforced lithium silicate.

Groups	N	Sum of Squares	df	Mean Square	F	Sig.	
							1.A Zirconia-Reinforced Lithium Silicate Post & Core	10	330244.438	2	165122.21	7.504	.003	
1.B-Zirconia-Reinforced Lithium Silicate One Piece Dowel Crown	10	594116.736	27	22004.324			
1.C-Zirconia-Reinforced Lithium Silicate Endocrown	10	924361.175	29				
Total	30						

Intra-group comparison of fracture resistance among cast metal restorations by one-way ANOVA showed no significant difference between the three different cast metal restoration groups (Table 3). Table 4 shows that the inter-group comparison using an unpaired t-test indicates a significant difference between ZLS post & core with crown (1.A) and cast post & core with crown (2.A) (p=0.054). Table 4 also shows a significant difference between ZLS one-piece dowel crown (group 1.B) and cast one-piece dowel crown (group 2.B) (p=0.003). However, there was no significant difference between ZLS endocrown (group 1.C) and metal endocrown (group 2.C). This indicates that the use of ZLS material as a post & core material in the premolar region will yield better results compared to cast post & core in the same region. Inter-group comparison using an unpaired t-test shows significant differences between ZLS post & core (1.A) and cast post & core (2.A). Table 4 also indicates a significant difference between ZLS one-piece dowel crown (group 1.B) and cast one-piece dowel crown (group 2.B). However, there was no significant difference between ZLS endocrown (group 1.C) and metal endocrown (group 2.C). This signifies that the use of ZLS material as a post & core material in the premolar region will give better results compared to cast post & core.

**Table 3:** Intra-group comparison of fracture resistance among Group 2.A, 2.B, and 2.C (Cast Metal) by one-way ANOVA.

Groups	N	Sum of Squares	df	Mean Square	F	Sig.
2.A-Cast Post & Core	10	76837.907	2	38418.954	1.774	.189
2.B-Cast One Piece Dowel Crown	10	584747.027	27	21657.297		
2.C-Metal Endocrown	10	661584.934	29			
Total	30					

**Table 4:** Inter-group comparison using unpaired t-test.

Groups compared	Mean difference	t value	df	P-value
Zirconia-reinforced lithium silicate post & core vs Cast post & core	127.8430000	2.006	18	0.054
Zirconia-reinforced lithium silicate one piece dowel crown vs Cast one piece dowel crown	-132.5110000	-2.308	18	0.003
Zirconia-reinforced lithium silicate endocrown vs Metal endocrown	71.94100	0.950	18	0.470

**Figure 9:**
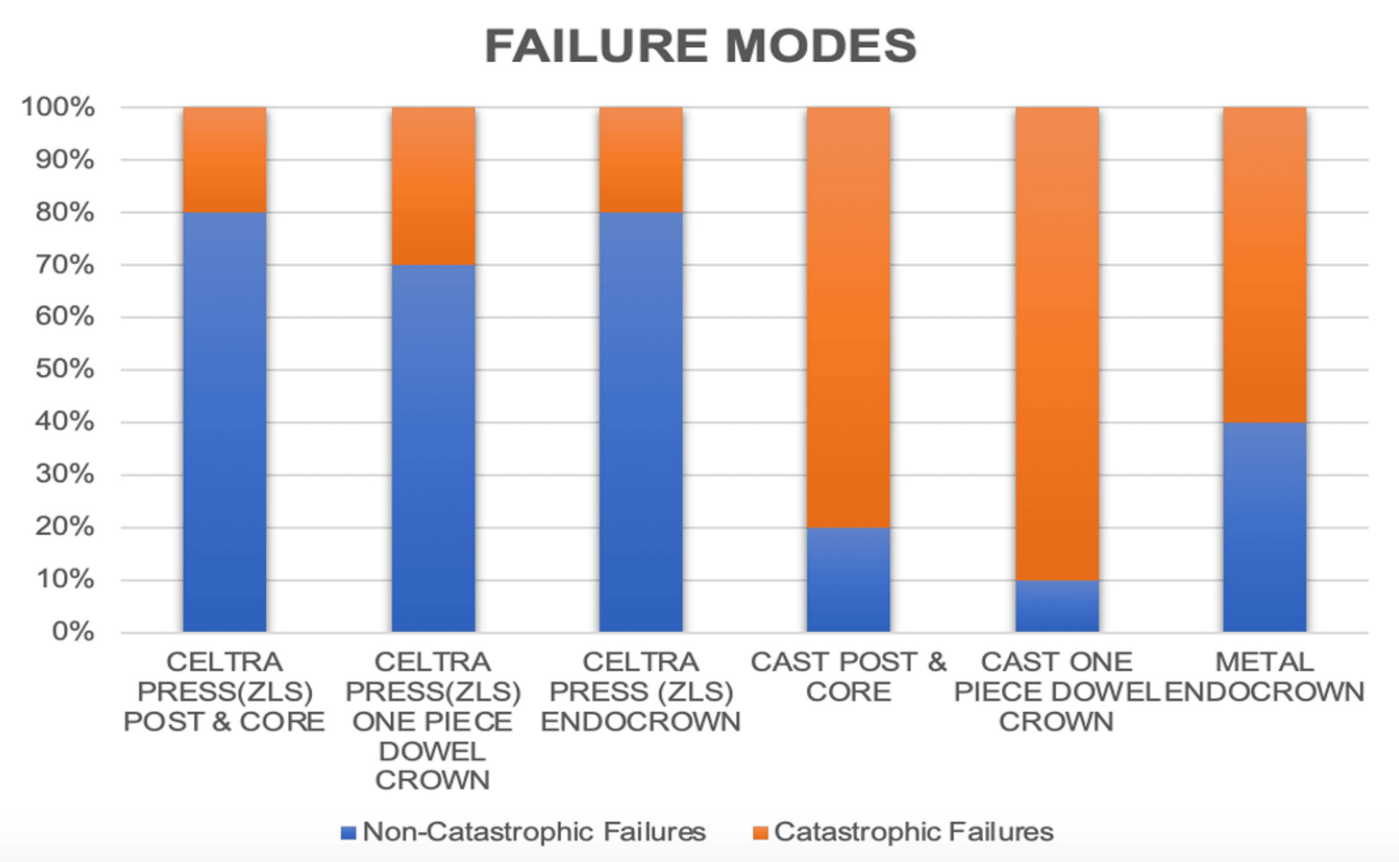
Percentage of catastrophic and non-catastrophic failures in each group.

The pictorial representation of the percentage of catastrophic and non-catastrophic failures in each group is represented graphically (Figure 9). Non-catastrophic failures are samples that had undergone fractures of teeth or restoration above the level of the CEJ. Catastrophic failures are samples that had undergone fractures of teeth or restoration below the level of the CEJ. The results of the study showed that ZLS (Groups 1.A, 1.B, & 1.C) restorations had a higher percentage of non-catastrophic failures (>70%) than catastrophic failures. It also showed that cast metal restorations (Groups 2.A, 2.B, and 2.C) had a higher percentage of catastrophic failures (>60%) than non-catastrophic failures.

## Discussion

Elsaka SE et al. (2016) [13] assessed the mechanical properties of ZLS glass-ceramic and concluded that Vita Suprinity ZLS glass-ceramic exhibited higher mechanical properties (fracture toughness, flexural strength, elastic modulus, and hardness) compared to IPS e.max CAD lithium disilicate glass-ceramic [13]. This new ceramic system consists of a lithium-metasilicate (Li2SiO3) glass-ceramic matrix that is reinforced with approximately 8-12% zirconium dioxide grains (ZrO2), presenting a fine-grained microstructure (Li2O-ZrO2-SiO2) after the crystallization process. Due to its increased translucency and color variety, ZLS can potentially be used to fabricate anatomical contours or monolithic restorations [9]. It is characterized by high strength (500 MPa) and excellent flow properties, therefore making it feasible for the fabrication of post and core systems with high strength along with excellent aesthetics. Therefore, in this study, ZLS fabricated post and core, one-piece dowel crown, and endocrown were compared with traditional cast metal for fracture resistance and failure modes [9]. Fernando Zarone did a literature review on this ZLS and concluded that ZLS is reported to be a biocompatible material, whose fracture resistance can withstand physiological chewing loads [14]. Research indicates that ZLS ceramics exhibit superior fracture resistance compared to several other restorative materials. This includes higher resistance to fracture than Polymer Infiltrated Ceramic Networks (PICN) and PMMA in occlusal veneers, greater fracture load than feldspar-based ceramics in tabletops, and superior fracture strength compared to PICN and hybrid composite resin in monolithic crowns. Furthermore, monolithic crowns made of ZLS demonstrated higher fracture resistance than bilayered, ceramic-veneered zirconia restorations [14]. Limitations of this material include it being the most difficult to machine among glass ceramics and its acid sensitivity. Due to its brittle nature and limited medium-term survival, its use for posterior bridges is not promising [15].

According to a study by Pîrvulescu IL et al. (2023), high-translucency ZLS obtained through the hot pressing technique provides optimal shade matching with adjacent teeth, sufficing as a good biomimetic material choice [16].

In this study, Celtra press (ZLS) blocks were used to fabricate post & core, one-piece dowel crown, and endocrown restorations on extracted mandibular premolars.

After testing these fabricated restorations in a universal testing machine, the fracture resistance and failure modes were analyzed. The result of this study shows that ZLS post and core showed high fracture resistance (610 N) compared to the cast post and core group (482 N), which was statistically significant. This may be attributed to the fact that the modulus of elasticity of ZLS (70 GPa) is closer to the modulus of elasticity of dentin (40 GPa) compared to cast metal restorations (208 GPa), and the stresses are dissipated more homogeneously in ZLS post & core restorations than in cast metal post and core restorations [17]. Sathyanarayana HP et al. (2012) assessed the maximum voluntary bite force in adults; the maximum bite force in the premolar region is 392 N and 601 N in the molar region [18]. This signifies that the use of ZLS material with a mean fracture resistance (610 N) can be used as a post & core material and will give promising results.

Regarding the restoration of endodontically treated anterior teeth, traditional cast metal restorations have disadvantages such as root discoloration, a blue-gray color effect at the cervical margins, and a risk of root fracture due to its high modulus of elasticity. Then, fiber posts were introduced, which showed high success in terms of aesthetics but lacked long-term chemical stability, resulting in failures of fiber posts. Zirconia posts came into use because of their high chemical stability, but because of their high elastic modulus (200 GPa), they resulted in root fractures [19]. With a modulus of elasticity of 70 GPa, far lower than that of zirconia posts (200 GPa), this novel ZLS material can solve this issue and result in fewer catastrophic failures. Bakke calculated that the average pressures acting on anterior teeth are 222 N [20]. The average occlusal stresses on the anterior teeth are less than the mean fracture resistance of the ZLS post & core and the one-piece dowel crown in this study, which are 610 N and 356 N, respectively. This is consistent with a 2020 study by Dogu OD et al. that examined the fracture strength of teeth that had undergone endodontic treatment and were restored using innovative CAD/CAM ceramic post systems. The study found that ZLS post restorations can tolerate the anterior bite force up to 200 N [19]. Unlike pure zirconia, ZLS can be etched, allowing strong adhesive bonding and improving its longevity in the oral environment. Its high fracture resistance, ability to be polished to a fine finish, and reduced wear on opposing teeth make it an ideal choice for various aesthetic applications and load-bearing situations in the mouth [16].

Regarding endocrowns, although the results of this study showed the mean fracture resistance of ZLS endocrown (450 N) is high compared to the mean fracture resistance of metal endocrown (378 N), the difference was not statistically significant. The mean fracture resistance of ZLS endocrown (450 N) is higher compared to the maximum bite force in the premolar region [18]. This implies that ZLS endocrown has adequate fracture strength to be used as a restoration for endodontically treated premolars. This is in accordance with the study by El Ghoul W et al. (2019), which found that the fracture resistance of ZLS endocrowns (Vita Suprinity) under axial and lateral loading was 2279 N and 1074 N on endodontically treated molars [21]. Thomas RM et al. (2020) conducted a systematic review comparing endocrown restorations on permanent molars and premolars, revealing identical success rates and no difference in the rate of endocrown failures between the two, implying that premolars may be suitable candidates for endocrowns [22]. Therefore, ZLS endocrowns appear to be a promising option for the restoration of severely damaged endodontically treated posterior teeth. Intragroup comparison between cast metal restorations showed no statistical difference among the three cast metal restorations, although cast post and core can still be used in clinical situations such as moderate to severe tooth loss (less than 2mm ferrule) and in situations where it is necessary to alter the angle of the core in relation to the tooth. Intragroup comparison between ZLS restorations using one-way ANOVA showed a significant difference among the three different ZLS groups (p < 0.05). Among ZLS restorations, the ZLS post & core showed superior fracture strength and should be preferred when clinical situations are suitable. ZLS endocrowns also showed promising results and can be used in clinical situations such as short clinical crowns and limited interocclusal space. Marginal gap measurements showed no significant differences between Zr and ZLS; however, ZLS had slightly better (higher) marginal adaptation [9].

In a recent study, Zoidis P et al. (2016) [23] proposed polyetheretherketone (PEEK) as an alternative framework material for endocrown restorations. They demonstrated that the elastic modulus of the PEEK framework (4 GPa) veneered with indirect composite resin could dampen the occlusal forces, protecting tooth structures better than ceramic materials. However, further long-term clinical evidence is required [23].

Regarding failure modes, they were assessed by careful visual inspection of the fracture lines throughout the tooth. Visual inspection of all fractured samples showed 23 non-catastrophic failures and 7 catastrophic failures among 30 ZLS restorations: 7 non-catastrophic failures and 23 catastrophic failures in cast metal restorations. This shows that cast metal restorations had higher catastrophic failures compared to ZLS restorations. This is mainly attributed to the factor modulus of elasticity, which is around 200 GPa for cast metal and 70 GPa for ZLS material. Therefore, it can be suggested that ZLS restorations can be a promising restorative material for the management of endodontically treated teeth. However, the machinability of ZLS is difficult and it is costlier than cast metal posts. Failure modes of ZLS restorations are predominantly non-catastrophic, which extends the lifetime of the teeth. ZLS material, combining superior mechanical properties of zirconia with superior aesthetics of lithium disilicate, has great potential to be used as post-endo restorations.

Limitations

The study lacks a scanning electron microscopic study to support failure results. Also, there is a need for comparison with other commonly used as well as advancing materials such as Zirconia, resin-based, PEEK, etc., to support the data.

## Conclusions

This in vitro study found that the ZLS post and core had the highest mean fracture resistance, as measured by the Universal Testing Machine, whereas the ZLS one-piece dowel crown had the lowest mean fracture resistance. The most common failure modes of ZLS restorations were non-catastrophic, whereas for cast metal restorations, catastrophic failures were the most common. ZLS restorations offer excellent aesthetic and mechanical properties with very few catastrophic failures, having great potential to be used as an alternative to cast metal restorations for the restoration of severely damaged endodontically treated teeth.
